# Relevance of interleukin-10RB to chronic hepatitis B virus infection and biological activities of interferon-λ and interleukin-22

**DOI:** 10.1007/s12072-012-9361-8

**Published:** 2012-03-14

**Authors:** Okki Cho, Jae Youn Cheong, Ka Jung Jun, Soon Sun Kim, Yong-Joon Chwae, Kyongmin Kim, Sun Park, Sung Won Cho

**Affiliations:** 1 Department of Microbiology, Ajou University School of Medicine, Youngtongku Wonchondong San 5, Suwon, 442-749 The Republic of Korea; 2 Department of Gastroenterology, Genomic Research Center for Gastroenterology, Ajou University School of Medicine, Youngtongku Wonchondong San 5, Suwon, 442-749 The Republic of Korea

**Keywords:** Chronic hepatitis B, HBV replication, IFN-λ, IL-10R2, IL-22, Single nucleotide polymorphism

## Abstract

**Purpose:**

The association of a single nucleotide polymorphism of interleukin (IL)-10RB codon 47 with the chronic hepatitis B virus (HBV) infection has been reported in two ethnic populations with different results, but not in a Korean population. IL-10RB is a subunit of receptor complexes for interferon-lambda (IFN-λ) and IL-22, which have antiviral and hepatocyte-protective activity, respectively. This study examined the association of IL-10RB K47E with the outcomes of HBV infection in Korean subjects and the cellular response to these cytokines.

**Methods:**

Genotypes of IL-10RB and the outcomes of HBV infection were analyzed in 1,000 Korean patients. The effect of IFN-λ or IL-22 on HBV replication and cell viability was assessed in hepatoma cells expressing IL-10RB K47 or E47. The transcript level of IL-10RB was examined in Epstein Barr virus-transformed B cells and hepatoma cells.

**Results:**

IL-10RB K47E was associated with chronic HBV infection but not with hepatoma in the Korean population. IL-10RB K47E was associated with the transcript level of IL-10RB in transformed B cells but not with the responses in hepatoma cells to IFN-λ or IL-22. HBV replication or 5-fluorouracil-induced cell death was suppressed by treatment of IFN-λ or IL-22 in an IL-10RB K47E-independent manner.

**Conclusions:**

IL-10RB K47E is related to chronic HBV infection in a Korean population, but not to cellular responsiveness to IFN-λ and IL-22.

**Electronic supplementary material:**

The online version of this article (doi:10.1007/s12072-012-9361-8) contains supplementary material, which is available to authorized users.

## Introduction

The interleukin (IL)-10RB subunit is shared by receptors for IL-10, IL-22, IL-24, IL-26, and interferon-lambda (IFN-λ) [1]. Although IL-10RB is expressed in almost all cells, these cytokines preferentially exert their functions in specific tissues because of the restricted expression of their specific receptor subunits [1]. IL-22R1 and IFN-λR (IL-28R1) are expressed in hepatocytes, whereas IL-10R1 and IL-28R1 are expressed in leukocytes [1, 2]. IL-22 enhances the hepatocyte survival in a T cell-mediated murine hepatitis model [3]. The antiviral effect of IFN-λ has been previously demonstrated with various viruses, including hepatitis B virus (HBV) and human hepatitis C virus [4, 5]. IFN-λ also affects cell viability and plays immunomodulatory roles in leukocytes [2, 6, 7].

HBV, a causative agent of chronic hepatitis leading to liver cirrhosis and hepatocellular carcinoma (HCC), preferentially replicates in hepatocytes [8]. HBV is a noncytopathic virus, and hepatocyte damage is not triggered by virus replication itself but by the cellular immune response to viral antigens [8]. HBV clearance is mediated by the immune response, and an insufficient immune response is regarded as a determinant for the development of chronic HBV infection [9]. Recently, the association of a cluster of class II cytokine receptor genes on chromosome 21q22 with susceptibility to chronic HBV infection was reported [10]. One gene in this locus is *IL*-*10RB*. Association of IL-10RB K47E with the outcome of HBV infection has been studied in two different populations with different results [10–12].

The present study investigated the association of IL-10RB K47E with the cellular responsiveness to IFN-λ and IL-22 and with the outcome of HBV infection in a Korean population. The IL-10RB K47E polymorphism did not affect the effects of IFN-λ or IL-22 on the HBV replication and cell viability despite the association of IL-10RB K47E with persistent HBV infection in a Korean patient population.

## Materials and methods

### Vectors and recombinant viruses

IL-10RB DNA in pEF2-IL-10R2 (kindly provided by Dr. Sergei Kotenko, New Jersey Medical School, Newark, NJ, USA) was subcloned into pLXIN (Clontech, Mountain View, CA, USA). An IL-10RB K47 allele expression vector was produced using a site-directed mutagenesis kit (Stratagene, La Jolla, CA, USA). *IL*-*10RB* DNA was subcloned into pREP4, an episomal expression vector (Invitrogen, Carlsbad, CA, USA). The leader sequences of *IL*-*10RB* were replaced by the leader sequences of Igκ in pSecTag2b (Invitrogen). Flag-tag sequences were inserted into the C termini of leader sequences. The inserted nucleotide sequences of all constructs were verified by sequencing analysis. Recombinant retroviruses were produced by the transfection of PT67 packaging cells with pLXIN-IL-10RB and purified by the centrifugation of the culture supernatant at 5,700*g* at 4 °C for 16 h.

### Cells

Hepatoma cell lines **(**Huh7 cells, HepG2 cells, Hep3B cells, SNU182, SNU354, SNU368, SNU387, and SNU449; Korean Cell Line Bank, Seoul, Korea) were used. Stable cell clones were produced by the infection of Huh7 cells with retroviruses expressing IL-10RB and then selected with G418 (1 mg/ml) and cloned. Semistable transfectants were produced by the transfection of Huh7 cells with pREP4-IL-10RB. All experiments using semistable transfectants were performed within 3 weeks of transfection after two rounds of hygromycin selection.

### Flow cytometric analysis

IL-10RB expression was analyzed by flow cytometry using an anti-IL-10RB antibody (R&D Systems, Minneapolis, MN, USA) and a phycoerythrin-conjugated antimouse IgG (H + L) antibody (eBioscience, San Diego, CA, USA) with FACS Canto (BD Biosciences, Rockville, MD, USA).

### Study subjects

From 2002 to 2008, 751 patients with chronic HBV infection and 249 spontaneously recovered subjects were enrolled from the Gastroenterology Department of Ajou University Hospital in Korea. The diagnostic criterion for chronic HBV infection was seropositivity for hepatitis B surface antigen (HBsAg) for more than 6 months. The study subjects were allocated to four groups according to their HBV infection status, clinical data, and serological profiles: (a) spontaneously recovered group, (b) chronic hepatitis group, (c) liver cirrhosis group, and (d) HCC group. The subjects in the spontaneously recovered group were defined as HBsAg negative, antibody to hepatitis B core antigen (anti-HBc IgG) positive, antibody to HBsAg positive, and having no evidence of liver disease. The subjects in the chronic hepatitis group had elevated ALT (≥2 times the upper limit of normal) at least once during the follow-up period and were HBeAg positive or HBV DNA positive. HBV DNA was detected by Digene Hybrid Capture II Assay (Digene Diagnostics, Beltsville, MD, USA) or the branched DNA assay (Versant™ 3.0; Bayer Healthcare LLC Diagnostic Division, NY, USA). The subjects in the liver cirrhosis group were diagnosed as having liver cirrhosis based on typical morphologic findings on computed tomography or ultrasonography and corresponding laboratory features or evidence of portal hypertension. The criteria for the diagnosis of HCC was based on imaging and an AFP level >400 ng/ml. In case of AFP <400 ng/ml, the diagnosis of HCC could be done by having two typical dynamic imaging studies for, or one typical imaging in, patients with cirrhosis, and a nodule >1 or 2 cm according to the Asia-Pacific Association of the Study of the Liver (APASL) guidelines. The subjects who were positive for anti-HBsAg and negative for anti-hepatitis B core IgG, positive for anti-human immunodeficiency virus, or who had other types of chronic liver disease were excluded from the study.

The general characteristics and clinical parameters of all the subjects are shown in Table 1. All the study subjects were Korean and therefore of a single ethnic population. Informed consent was obtained from each subject, and the Institutional Review Board of Human Research of Ajou University Hospital approved the study protocol.

**Table 1:** General characteristics of the study subjects

Characteristics	SR (*n* = 249)	CH (*n* = 295)	LC (*n* = 201)	HCC (*n* = 255)
Gender (% M/F)	77.2/22.8	72.7/27.3	75.9/24.1	76.4/23.6
Age	44.1 ± 5.4	38.0 ± 9.1	46.2 ± 10.4	53.6 ± 10.3
AST (U/l)	31.9 ± 53.7	83.4 ± 121.3	75.0 ± 79.5	86.7 ± 100.2
ALT (U/l)	38.9 ± 28.9	113.1 ± 152.8	72.3 ± 99.1	50.8 ± 46.9
Total bilirubin (mg/dl)	0.9 ± 1.0	1.0 ± 1.6	2.1 ± 3.3	2.5 ± 11.2
Albumin (g/dl)	4.4 ± 0.2	4.2 ± 0.3	3.7 ± 0.7	3.6 ± 0.6

*SR* spontaneously recovered subjects, *CH* chronic hepatitis B, *LC* liver cirrhosis, *HCC* hepatocellular carcinoma

### Genotyping

Genotyping was performed using a GoldenGate genotyping assay kit (Illumina Inc., San Diego, CA, USA) in accordance with a standard protocol and the Sentrix array matrix chip (Illumina Inc.). The chip was imaged using a BeadArray Reader (Illumina Inc.). Genotyping analysis was performed using Illumina’s BEADSTUDIO software (Version 3.0.22).

### HBV core DNA isolation

HBV core DNA was isolated as described previously [13]. Huh7 cells were cotransfected with 500 ng of pEGFP-N1 (Clontech) and 2.5 μg of pPB [13] using Lipofectamine 2000 (Invitrogen) and harvested 48 h after transfection. Twenty percent of cells were subjected to FACS analysis to determine the transfection efficiency. Viral core particles were isolated from the rest of the cells, and the HBV DNA was extracted from core particles.

### Real-time PCR and semiquantitative reverse transcription-PCR

We performed real-time PCR for HBV DNA as described [14, 15]. For real-time reverse transcription (RT)-PCR of the IL-10RB transcript, we used a SYBR Green mixture (Applied Biosystems, Foster City, CA, USA) and IL-10RB-specific primers (TaKaRa Bio, Otsu, Japan). Semiquantitative RT-PCR for IL-10RB was performed using 5′-GGCTGAATTTGCAGATGAGCATTC-3′ and 5′-GAAGACCGAGGCCATGAGG-3′. The band intensity of the PCR product was measured with a densitometer (Gel Logic 200 Imaging System; Kodak). The data were normalized to GAPDH.

### Cell survival assay

The viability of cells was measured with a 3-[4,5-dimethylthiazol-2-yl]-2,5-diphenyltetrazolium bromide (MTT) assay (Sigma). Cells were treated with 200 μM of 5-fluorouracil (5-FU), an inhibitor of DNA synthesis, at 37 °C for 2 days and then subjected to the MTT assay. Cell viability was calculated as the percentage of the sample optical density (OD) relative to the OD of uninfected cells. The increment of viability was calculated as the difference between the viabilities in the presence and absence of the indicated cytokine.

### Immunoblotting

STAT3 in cells was detected by immunoblotting using antibody specific to STAT3 (Cell Signaling, Danvers, MA, USA).

### Luciferase reporter assay

Six tandem repeats of the STAT3 responsive element were cloned into pGF1-mCMV, a lentiviral reporter vector (System Biosciences, TR010PA-1). Cells were infected with reporter lentivirus and 2 days later were treated with the indicated cytokines. Luciferase activity was measured using luciferin (Promega, Madison, WI, USA) and a luminometer (Molecular Devices, Sunnyvale, CA, USA).

### Statistical analysis

For univariate analysis, the χ^2^ test was used to analyze the Hardy–Weinberg equilibrium of alleles at individual loci and the independent sample *t* test was used to analyze normally distributed continuous variables. Odds ratios with 95 % confidence intervals were computed by logistic regression using SPSS version 11.0 software (SPSS, Chicago, IL, USA). The odds ratios were adjusted for age and sex as covariables. For all the statistical tests, significance was set at *P* < 0.05.

## Results

### Association of IL-10RB K47E with chronic HBV infection, but not with HCC, in Korean subjects

The association of IL-10RB K47E (rs2834167) with chronic HBV infection was studied in 194 sibling pairs from the Gambia (the relative frequency for K47 allele was 93.9 %) [10] and 682 Chinese Han subjects (the relative frequency for K47 allele was 58.7 %) [12]. Similar examinations have not hitherto been done in Korean populations. Presently, the association of IL-10RB K47E with outcomes of HBV infection was assessed in 1,000 Koreans, including 751 patients with chronic HBV infection (including liver cirrhosis and HCC) and 249 patients who had spontaneously recovered from HBV infection. The relative frequency of K47 allele in the controls was 41 %, which was compatible with the frequency in 934 Japanese volunteers in a previous study [16]. IL-10RB K47 was associated with an increased risk of a chronic HBV infection in the codominant model (Table 2). However, there was no significant association of IL-10RB K47E with HCC development (Table 3). These findings support the suggestion that the risk factors for the development of HCC are different from those for chronic HBV infection.

**Table 2:** Analysis of the association of an *IL*-*10RB* polymorphism (rs2834167) with chronic HBV infection versus self-limited HBV infection

Allele	Codominant		Dominant		Recessive	
	OR (95 % CI)	*P* value	OR (95 % CI)	*P* value	OR (95 % CI)	*P* value
A>G	1.25 (1.01, 1.54)	0.037	1.14 (0.68, 1.33)	0.087	1.18 (0.97, 1.42)	0.092

Genotype distributions and *P* values are shown for logistic analysis of three alternative models controlling for age and sex as covariates

*OR* odds ratio, *CI* confidence interval

**Table 3:** Analysis of the association of an *IL*-*10RB* polymorphism (rs2834167) with HCC versus chronic HBV infection without HCC

Allele	Codominant		Dominant		Recessive	
	OR (95 % CI)	*P* value	OR (95 % CI)	*P* value	OR (95 % CI)	*P* value
A>G	1.084 (0.849, 1.385)	0.517	0.934 (0.771, 1.131)	0.486	1.231 (0.991, 1.528)	0.060

Genotype distributions and *P* values for logistic analysis of three alternative models controlling for age and sex as covariates are shown

*OR* odds ratio, *CI* confidence interval

### Association of IL-10RB K47E with its transcript levels in B cells, but not in hepatoma cells

**Fig. 1:**
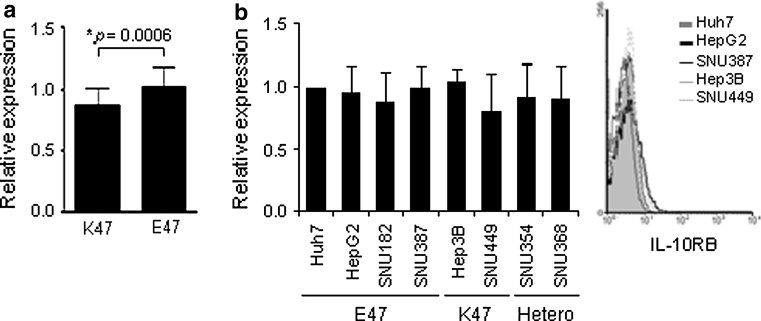
The IL-10RB expression levels in B cells and hepatoma cells. The IL-10RB transcript was determined by real-time RT-PCR (**a**) or semiquantitative RT-PCR (**b**) and surface protein levels were determined by flow cytometry (**b**). **a** EBV-transformed human peripheral B cell lines. The number of tested cell lines was 7 for K47 and 12 for E47. **b** Hepatoma cell lines with known IL-10RB genotype. Data are shown as the mean ± standard deviation of three to five independent experiments. *Student’s *t* test

Surface and mRNA levels of IL-10RB are higher for the E47 variant than the K47 variant [10]. Consistent with this, significantly lower transcription of IL-10RB K47 than E47 was observed in Epstein–Barr virus (EBV)-transformed B cells derived from homozygous Korean individuals (Fig. 1a; *P* < 0.001). However, similar levels of IL-10RB expression were observed in hepatoma cell lines regardless of their genotype (Fig. 1b).

### IL-10RB K47E does not affect HBV replication in response to IFN-λ and IL-22

**Fig. 2:**
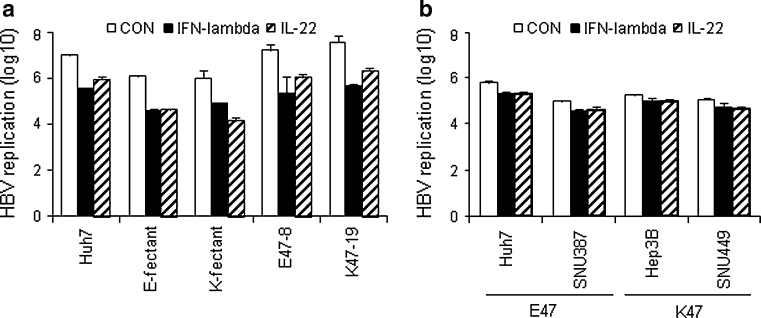
IL-10RB K47E is not associated with the effect of IFN-λ and IL-22 on HBV replication. Cells were transfected with pPB, an HBV plasmid, together with pEGFPN1 (1/5 of the amount of pPB) for normalization of transfection efficiency. 1 day later, the cells were treated with 100 ng/ml of IFN-λ or IL-22 for 24 h. HBV core DNAs were isolated from these cells and subjected to real-time PCR for quantification of HBV DNA copy numbers. **a** Cells containing episomally maintained vectors expressing IL-10RB E47 (*E-fectant*) or K47 (*K-fectant*) and Huh7-derived stable clones expressing either IL-10RB E47 (*E47-8*) or K47 (*K47-19*). *CON* without cytokine treatment. **b** Hepatoma cell lines. Data represent the mean ± standard deviation of five replicates

HBV replication in hepatoma cells expressing IL-10RB K47 or E47 in response to IFN- λ or IL-22 was determined to explore whether the single nucleotide polymorphism of an IL-10RB extracellular domain affects the cellular response to IL-22 and IFN-λ. First, Huh7 cells were transfected with episomally maintained expression vectors for each IL-10RB allele, and then IL-10RB mRNA and surface protein levels were analyzed (Supplementary Fig. a). Also, IL-10RB expression in Huh7 cells stably transfected with retroviral expression vectors for IL-10RB was verified by real-time RT-PCR (Supplementary Fig. b). IL-10RB transcript and protein levels were higher in cells with IL-10RB expression vectors than in control cells, but were not affected by genotype. These cells were then transfected with HBV plasmid and 24 h later were treated with IFN-λ or IL-22 for another 24 h. HBV replication was analyzed by real-time PCR. HBV replication was inhibited both by IFN-λ and IL-22 in all these cells (Fig. 2a). However, IL-10RB K47E did not affect the antiviral effect of these cytokines. Next, HBV replication was compared in four hepatoma cell lines. HBV replication was slightly reduced in these cells in response to IFN- λ and IL-22, but the decrease was not associated with IL-10RB K47E (Fig. 2b). These results supported the suggestion that IL-10RB K47E does not directly affect IFN-λ- or IL-22-mediated suppression of HBV replication in hepatocytes.

### IL-10RB K47E does not influence the effect of IFN-λ and IL-22 on cell viability

**Fig. 3:**
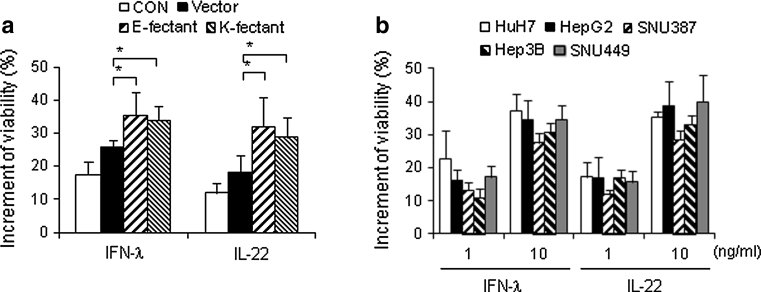
Upregulation of cell survival by IFN-λ and IL-22 is not associated with IL-10RB K47E. Cells were treated with 200 μM of 5-FU in the presence or absence of the indicated cytokine (10 ng/ml) at 37 °C for 2 days. The cell viability was analyzed by a MTT assay. *E-fectant*, *K-fectant* Cells containing episomally maintained vectors expressing IL-10RB E47 or K47. *HuH7*, *HepG2*, *SNU387* Hepatoma cell lines with IL-10RB E47. *Hep3B*, *SNU449* Hepatoma cell lines with IL-10RB K47. Data represent the mean ± standard deviation of five replicates. *P < 0.05, Student’s *t* test

Although HBV is not a cytotoxic virus, hepatocyte death occurs as a result of the cell-mediated immune response to the HBV antigen. Given that IL-22 promotes hepatocyte survival in a T cell-mediated murine hepatitis model [3] and IFN-λ also affects cell viability [17], it was appropriate to examine the influence of IL-10RB K47E on cell survival in response to IFN-λ and IL-22 when cell death was induced by treatment with 5-FU, a chemotherapeutic agent (Fig. 3). When cells were treated for 48 h with 5-FU, viability was increased in the presence of IFN- λ or IL-22, but this change was not correlated with genotype. Notably, populations of cells overexpressing IL-10RB were significantly more viable than controls. These results imply that the IL-10RB K47E does not affect hepatocyte response to IFN-λ and IL-22.

### STAT3 activation is involved in the effect of IFN-λ and IL-22 on cell viability

**Fig. 4:**
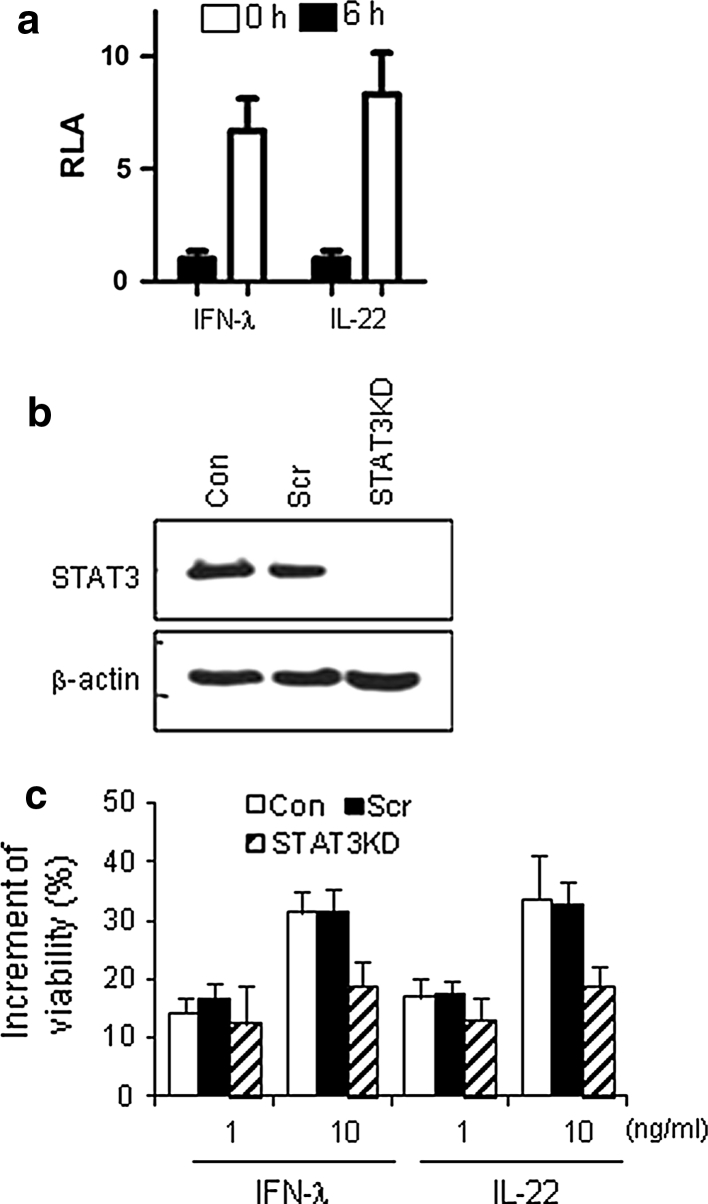
IFN-λ enhances cell survival through STAT3 activation. **a** STAT3 activation in Huh7 cells was monitored by a luciferase reporter assay. Data are shown as the mean ± standard deviation of two independent experiments performed in triplicate. **b** STAT3 knock-down was assessed by immunoblotting in Huh7 cells (*CON*) and cells transfected with a shRNA-expressing vector for a nonspecific target (*Scr*) or specific to STAT3 (*STAT3KD*). **c** The viability of cells treated with 200 μM 5-FU in the presence or absence of the indicated cytokine at 37 °C for 2 days was analyzed by a MTT assay. Data are shown as the mean ± standard deviation of five independent experiments

The cell-protective effect of IFN-λ was unpredicted. Thus, to support the above results, the involvement of STAT3 activation in IFN-λ-mediated cell survival was assessed, since it was revealed that STAT3 plays a critical role in IL-22-mediated cell survival and that IFN-λ activates STAT3. First, STAT3 activation was analyzed using a luciferase reporter assay (Fig. 4a). Luciferase activity was increased by IFN-λ treatment of cells. Next, STAT3 expression was knocked down in Huh7 cells using siRNA specific to STAT3 (Fig. 4b), and then the cell viability of these cells was examined after treatment with IFN-λ or IL-22 (Fig. 4c). The increment of cell viability was higher in control cells (untransfected or control siRNA-transfected cells) by 10 ng/ml of cytokine treatment than that in STAT3KD cells. These results imply that IFN-λ upregulates cell viability through STAT3 activation.

## Discussion

Presently, the association of IL-10RB K47E with HBV persistence was demonstrated in Korean subjects. However, IL-10RB K47E did not affect the cellular response to IFN-λ and IL-22. IL-10RB K47E affects responses to IL-10 because of differences in the expression level between IL-10RB K47 and E47 [10]. However, a reduced responsiveness to IL-10, an immunosuppressive cytokine, in IL-10RB K47 could not account for its association with chronic HBV infection. Hence, the effect of this SNP on cellular responsiveness to IFN-λ and IL-22 capable of suppressing HBV replication and increasing hepatoma cell viability was examined. Although no direct influence of this SNP on the cellular response to these cytokines was observed, the cellular responsiveness was dependent on IL-10RB expression level and a lower expression of IL-10RB K47 than E47 was shown in B cells. We do not know the reason for the lack of association of IL-10RB K47E with IL-10RB expression level in hepatoma cell lines, but the limited number of hepatoma cell lines we analyzed may be one factor. Alternatively, this SNP may be indirectly associated with IL-10RB expression level. Concordant with this speculation, a recent report described the linkage disequilibrium of IL-10RB K47 with a SNP (rs999788C) in the IL-10RB promoter lacking a NF-κB binding site [16]. In that case, IL-10RB K47E may indirectly affect the cellular response to IFN-λ and IL-22. This study focused on the association of IL-10RB K47E with the effects of IFN-λ and IL-22 in cultured hepatoma cells. Thus, the results may be different in the human body, where cytokines binding to IL-10RB affect hepatocytes directly and indirectly through modulation of immune cells.

The present results demonstrate the suppressive activity of IL-22 and IFN-λ on HBV replication. In contrast to IFN-λ, antiviral activity of IL-22 has not been well documented. However, compatible with the present findings, a recent report demonstrated that IL-22 shows weak inhibitory effect on HBV replication in murine hepatocytes [18]. In that report, the transcriptional program activated by IL-22 resembled more the response induced by IL-6 rather than the INF-λ-mediated response [18]. It is notable that IL-6 suppresses HBV replication through the inhibition of nucleocapsid formation and of two transcription factors, hepatocyte nuclear factor 1α and 4α, which are essential for HBV gene expression and replication [19, 20]. Thus, the current results suggest antiviral activity of IL-22 on HBV in human cells. It will be interesting to find out the suppressive mechanisms of IL-22 on HBV replication.

We showed the protective activity of IL-22 and IFN-λ on chemotherapy-induced hepatoma cell death. The protective effect of IL-22 is consistent with a previous report that IL-22 contributes to the chemotherapeutic resistance of human lung cancer cells through the activation of STAT3 [21]. Also, the aberrant expression of IL-22R1 and autocrine IL-22 stimulation contribute to tumorigenicity in anaplastic large cell lymphoma [22]. However, the present finding of a cell-protective effect of IFN-λ is unexpected, in light of the report that IFN-λ inhibits the proliferation of T lymphoma cells expressing IL-28R [17]. Comparable with our results, though, IFN-β shows contradictory effects on cell proliferation and apoptosis depending on the dose [23]. Also, type 1 IFNs can stimulate cell survival or apoptosis in myeloma cells depending on experimental conditions [24–26]. As regulators of IFN-α-induced cell survival, several molecules, such as G1P3 and USP18, are recognized [27, 28]. In line with our results only three lines among nine human esophageal carcinoma cell lines were found to be sensitive to the antitumor effects of IFN-λ despite uniformly increased IFN-λ-induced antiviral gene expression [29]. The current findings provide evidence that not only IL-22 but also IFN-λ can stimulate cell survival through involvement of STAT3 activation.

Participation of IL-10RB in STAT3 activation has been previously suggested by the observations that STAT3 is activated in cells transfected with both IL-10RB and IL-22R1 or with both IL-10RB and IL-28R1, but not with either receptor alone [30, 31]. STAT3 tyrosine phosphorylation occurs through an interaction of STAT3 with the IL-22R C terminus [32]. IFN-λ-induced STAT3 tyrosine phosphorylation has not been well studied, except that it seems to be independent of IL-28R1 tyrosine residues 343 and 517 [17]. How the IL-10RB affects STAT3 tyrosine phosphorylation requires further study.

In conclusion, the study results suggest that IL-10RB K47E is associated with persistent HBV infection in a Korean population, but not with cellular responsiveness to IFN-λ as well as IL-22.

## Electronic supplementary material

Below is the link to the electronic supplementary material.

Supplementary material 1 (TIFF 57 kb)
