# Supplementary figures and images for: Relevance of interleukin-10RB to chronic hepatitis B virus infection and biological activities of interferon-λ and interleukin-22
Source: Hepatol Int. 2012 Mar 14;7(1):111–8. doi: 10.1007/s12072-012-9361-8 (PMC3601266; doi:10.1007/s12072-012-9361-8)

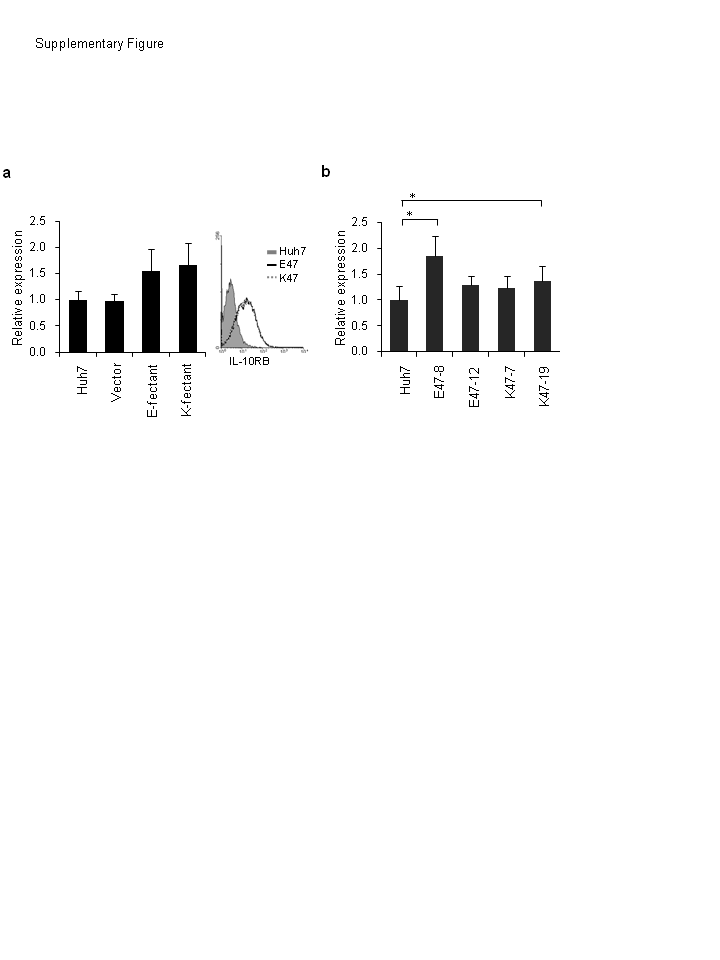

Supplement: Supplementary file 1 — Supplementary material 1 (TIFF 57 kb) [file 12072_2012_9361_MOESM1_ESM.tif]
